# Beyond a buzzword: the need for shared language, education, and enhanced reporting of disability inclusive co-design research

**DOI:** 10.1186/s40900-025-00831-y

**Published:** 2026-01-26

**Authors:** Kelsey Chapman, Joyce Yi, Michael Norwood, Kelly Clanchy, Joan Carlini, Camila Shirota, Maretta Mann, Jessie Mitchell, Elizabeth Kendall

**Affiliations:** 1 Reimagining Disability, Griffith University, Parklands Dr, Southport, QLD 4222 Australia; 2 The Hopkins Centre, Griffith University, Nathan, QLD 4111 Australia; 3 The School of Allied Health, Sport and Social Work, Griffith University, Gold Coast, 4222 Australia; 4 Department of Marketing, Griffith University, Parklands Dr, Southport, QLD 4222 Australia

**Keywords:** Inclusive research, Disability, Co-design, Research management, Patient and public involvement

## Abstract

**Background:**

Co-design is an increasingly prominent method for achieving disability-inclusive research. Co-design draws attention to the value of involving people with lived experience of disability in shaping research questions, methods, and outcomes, making it an ideal vehicle for inclusion. Yet, the rise in popularity of co-design in disability research has created challenges. Inconsistent definitions, misaligned practices, and inadequate reporting have undermined both the rigour of the method and its potential to generate meaningful and impactful outcomes.

**Discussion:**

In this commentary, we argue that safeguarding the future of disability inclusive co-design requires a systemic shift towards accountability and transparency. We propose four urgent recommendations to enhance and maintain the integrity and impact of co-design: 1. Establish a shared language that distinguishes levels of participation, clarifies epistemic assumptions, and articulates underlying values. This is critical to ensure that co-design claims accurately reflect the nature of involvement. 2. Build capacity and capability for researchers and people with lived experience of disability. This includes in facilitation skills, ethical engagement, inclusive communication, and navigating power dynamics, to avoid tokenism and collaborate more effectively. 3. Develop a co-design-specific reporting framework to make relational, procedural, and decision-making processes more transparent. 4. Mandate better reporting of co-design practices by funders, journal editors, and institutions to ensure accountability and reportable rigour of co-design research with people with disability.

**Conclusion:**

Ultimately, co-design can either be a powerful tool for inclusive research or a hollow promise that perpetuates the exclusion it seeks to remedy. Adopting these recommendations for language, capacity, and standard, mandated reporting are essential steps for safeguarding the integrity of the method. It will also support more accountable, authentic, and impactful research that truly reflects the voices, priorities, and lived experiences of collaborators with disability.

## Abstract (plain language summary)

Co-design is a way of doing research where people with disability work with researchers to create, carry out, and share research. This method is now very popular. People with disability should have a say about how and what research is done. But the word “co-design” is sometimes used too often without really involving people with disability. In this paper, we talk about four important things that need to be improved to make co-design better. First, we need to use clear language, so everyone understands co-design and how it is different from other types of research. Second, everyone who does co-design research needs proper training and support to work together. Third, we need better ways to report what was done in each study, who was involved, how choices were made, and how people with disability were included. Fourth, research publications, funding agencies, and universities should ask for this reporting from researchers so that co-designed research can be trusted. By improving how co-design is done and reported, we can make sure it leads to more inclusive, fair, and helpful research that meets the needs of people with disability.

## Background: the rise of co-design

In recent years, the concept of co-design has emerged as a prominent approach in disability inclusive research, drawing upon a rich and long history of collaborative and participatory methodologies that have evolved from multiple disciplines [1–3]. The roots of co-design lie in product development, reflecting the need to engage with end-users throughout the process to ensure solutions are both useable and acceptable to customers [2, 4]. Although the term “co-design” suggests an approach to research that prioritises the involvement of end-users to develop a solution to a problem, the reality is often more complex, involving partnerships between multidisciplinary stakeholders (“co-designers” or “co-researchers”).

In this paper, we argue that the term co-design is poorly understood and inappropriately overused in disability research, serving as an artificial umbrella for a variety of collaborative and transformative research methods that aim to promote inclusion. This inappropriate application of the approach risks undermining the integrity of the disability inclusion movement and highlights a critical gap: despite the growing emphasis on participation, there is limited guidance on how to implement co-design rigorously and meaningfully. By focusing on disability research, we address an underrepresented domain where co-design has genuine transformative potential, offering insights into inclusive practices that can strengthen both methodology and outcomes. Additionally, the widespread inadequacy in reporting methods and approaches hinders meaningful assessment of the research’s inclusivity and veracity. To drive innovation in disability inclusive research in general and co-design specifically, we advocate for four ways forward, which include: (1) developing a shared language, (2) building the capacity and capabilities of co-designers, (3) developing a co-design specific reporting framework, and (4) mandating reporting of co-design methods and outcomes.

This perspective is informed by years of collaborative research, including co-design with people with disability across multiple projects and contexts. These experiences, including personal lived experience of disability, chronic health conditions, and caring for people with disability, have shaped our understanding of co-design as both a methodological and relational practice. Our inclusive research work, spanning more than 30 professional years combined, has occurred in marketing, social policy, disability and rehabilitation, healthcare, public transportation, psychology, and rehabilitation engineering, and has shaped our perspectives and discussion of the future of co-design in disability research.

## Main text: a way forward

### Build a shared language

The interpretation and implementation of co-design vary widely across research contexts and fields [5]. While co-design has gained significant traction in disability research, particularly in Australia where the authors based, its principles are widely applicable across sectors, including environmental sustainability, social marketing, public health, education, and service design. This broader relevance reinforces the need for a shared language that can be adapted to different disciplinary, geographic, and cultural contexts. Even within the authorship team and our internal collaborations, we have debated the definition of co-design, with our interdisciplinary backgrounds shaping how we apply co-design practices into our own disability research.

Co-design has been used to describe activities along the research engagement spectrum [6] from user observation, informing, consultative interviews, and surveys, to more empowered partnerships involving deep collaboration between co-designers through shared governance, conduct, and dissemination of research [5]. Many published studies employ the term co-design when other terminologies may be more appropriate [7]. Misuse of terminology can lead to superficial engagement for people with disability being inappropriately labelled as disability-led knowledge [8]. Terms such as participatory research, inclusive research, patient and public involvement, and lived experience are often used interchangeably or without clear definition. We argue that these terms should be co-defined with collaborators to reflect the values and contexts of projects, although there should be some shared clarity on what these terms mean for research rigour. Importantly, co-design does not inherently lead to emancipatory or transformational outcomes despite common assumptions [1, 7]. Without clearly defined epistemic values, embedded implementation strategies, and transformation goals, co-design may fall short of delivering the desired social change [9, 10]. This situation risks undermining the impact of co-design and could reduce long-term support for co-design methods [11].

Furthermore, co-design in cross-cultural settings reveals the impact that power differentials may have on participation and decision-making, reinforcing the need for context sensitive language and design [12]. These nuances and diversities of co-design highlight that co-design is not a one-size-fits-all methodology, but a relational and situated practice that should be shaped by the contexts in which they are embedded, while retaining methodological rigour [13]. Therefore, addressing these persistent issues is essential to ensure meaningful consumer input is both valued and sustained in research, while providing clarity and flexibility across contexts.

### Develop the capacity of co-designers

Poor operationalisation of co-design can demand substantial energy and time commitments from co-designers, without a corresponding guarantee of meaningful involvement, respect for their knowledge, or improved outcomes. Without formal training, co-designers, including researchers, are often underprepared to manage the complexities of inclusive research. To address the variation in how co-design is understood and practiced, we must invest in education and capacity building for all co-designers, including researchers who facilitate collaboration [3, 4]. In our own research, the early stages of engaging in co-design work felt chaotic and challenging. We often learned through trial and error discovering what worked well, identifying barriers and challenges, and understanding the kinds of support and resources we needed. Greater access to capacity building and training would have t strengthened our work, making it more rigorous, inclusive, and better structured. Such support is particularly important for early career researchers and higher degree research candidates.

Researchers must be supported to assess how they plan, apply, and interpret co-design practices, including recognising the varying levels of involvement, collaboration, and transformation required for different challenges [2, 7]. Researcher capacity for co-design includes a combination of conceptual, relational, and practical competencies. Conceptually, researchers must understand participation as a continuum and be able to justify the level of involvement they adopt [4]. Researchers need to be able to define and describe the underlying mindset (or way of knowing) that drives their co-design practices, as this overarching ontology and epistemology ultimately determine authentic inclusion [3, 7, 11]. Relationally, they need to build trust, share power, and navigate tensions that may arise when diverse perspectives challenge traditional research norms [10, 19]. Practically, researchers must uphold effective facilitation skills, inclusive communication, ethical engagement, and cultural competence to ensure the process is safe and accessible for all participating [10].

Building capacity also requires recognising the differentiated needs of co-design participants with different disabilities and access requirements across contexts. Researchers must have the skills to be responsive to the diverse and differentiated needs of people with disability [21, 22]. Inclusive principles and methods need to be adapted to suit varying access requirements, communication styles, and supports, which requires a unique skill set [21, 22]. For example [21, 22]. For example, co-design with people with disability may require additional extended timelines, Easy Read materials, and tailoring to support the importance of flexible and context responsive approaches [23, 24]. Researchers must be adept in planning and responding to structural barriers of their contexts, including limited funding, compressed timelines, inaccessible systems, and resource inequities, particularly in the Global South. Addressing these barriers requires not only training but also systemic advocacy for change. While systemic change is frequently advocated, it is important to recognise that individuals and communities experiencing poverty have limited capacity to alter entrenched structural conditions on their own. Sustainable transformation, therefore, requires collective and coordinated action through partnerships between communities, researchers, and institutions, which are supported by policies that redistribute resources, amplify lived experience, and build long-term capacity [25].

Additionally, researchers must also engage in reflexive practice, critically reflecting on their positionality, assumptions, and the evolving dynamics of the co-design process [1, 7]. Finally, capacity includes the ability to embed co-design within institutional structures, advocate for adequate time and resources, and demonstrate the scientific rigour and impact of these collaborative approaches [10, 11].

### Develop a co-design-specific reporting framework

Without consistent reporting, co-design in research can lack transparency and allow tokenism. Within our authorship group, we use diverse tools to report on our co-design work, including the Guidance for Reporting Involvement of Patient and Public (GRIPP2) [27, 28], International Association for Public Participation scale (IAP2), and other supplementary materials. Such tools are designed to improve reporting quality for inclusive research by prompting researchers to document what involvement occurred, how, and with what impact [29]. While the GRIPP2 and similar tools have been important in progressing the reporting of public and patient engagement in research, they lack clear mechanisms for reporting on the relational dynamics and conditions for creating authentic collaboration rather than tokenistic or surface consultation [30].

Furthermore, after conducting several literature reviews on co-design, we have been left questioning how other researcher implemented their co-design studies and what factors most influenced or transformed their approaches to achieve greater rigour and inclusivity. Historically, there has been no co-design specific reporting framework or requirements consistently used to enhance rigour. The recently published Co-Design Evaluation Framework, designed to consider the impact, structures, and contexts to support enhanced planning and evaluation for co-design, advances reporting considerations for rigour [31]. However, this Framework has not yet been widely tested or adopted within disability research.

To support genuine accessibility and inclusion, we call for a tailored reporting tool or an extension of existing tools collaboratively developed through co-design [3, 12, 32]. This tool must ensure reference to: the depth, scope, and nature of co-designer involvement; the influence of co-designers on research decision-making; the representation and outreach strategy used to involve the ‘harder-to-reach’ communities and individuals [32, 33]; the types of co-design methods and approaches used; whether support and training were provided; and the length of engagement and collaboration [18].

It must describe the unique relational dynamics and power-sharing processes and balance flexibility with rigour, allowing for diverse applications of co-design while ensuring clear reporting on methods [11, 16, 19, 20, 26, 34] and reflect on whether the process meaningfully facilitated equitable knowledge production [19, 20, 26, 35]. It must ensure the articulation of tangible outcomes for co-designers, such as capacity building, policy change, or long-term partnerships [8]. It is also critical that co-design retains global applicability, meaning that reporting tools must be adaptable to low-resource settings and culturally diverse contexts. Co-design in areas like the Global South often requires specific planning to navigate unique contextual challenges like limited digital and physical infrastructure, diverse languages, and differing conceptions of knowledge and participation. Including geographically diverse perspectives and acknowledgement of additional low-resource challenges within reporting tools will help ensure that co-design practices are inclusive not only in intent but also in implementation and reporting.

Further, any newly developed reporting mechanisms should include an evaluation component where co-designers themselves evaluate the process to ensure the output reflects lived experience, values, and priorities – thereby reinforcing the relational and ethical integrity of co-design as a practice [5]. The transparency, rigour and consistency of the growing co-design field depends on the accurate and consistent reporting of these elements. While pockets of good practice exist, the lack of consistent reporting makes it difficult to verify and compare co-design approaches across studies. This underscores the urgency of developing tools that support transparency and enable meaningful case-based learning.

### Mandate reporting of co-design activities

Without clear expectations from funders and publishers about reporting of co-design practices, and without mechanisms to evaluate reporting quality, existing and new tools risk being adopted sporadically or not at all [3, 5, 8, 15, 24]. Consequently, funders and editors should take a more active role in supporting robust and ethical co-design reporting [11, 12]. This role could involve requiring detailed representation and outreach strategies and co-design methodology plans [3, 13, 14], as well as identifying tools that enable active participation while addressing power imbalances [1, 2, 12]. Mandatory reporting requirements could include outlining researcher training and capability in co-design [15, 20, 21] and tracking of tangible outcomes for co-designers – such as authorship, remuneration, and participation in the decision-making through the use of validated reporting tools [23]. While mandated reporting can improve transparency, it is important to note that it could disadvantage under-resourced teams or those working in low-resource settings. Reporting frameworks should be scalable and adaptable to different contexts and circumstances to support rigour, while avoiding reinforcement of inequities and exclusion of resource-constrained co-design.

As co-design methodologies continue to mature, the tools that guide their reporting must evolve in parallel. Equity must be embedded not just in outcomes, but in the process of knowledge production itself. This requires a shift in mindset where co-design is not owned by researchers but is a participatory approach guided by shared governance with diverse partners. Such a model actively redistributes power by granting communities and end-users meaningful authority in decision-making, from setting the research agenda to defining its impact [5, 16, 36]. Within this way of working, reporting is transformed from a compliance exercise into a vital mechanism for accountability to all co-designers and broader partners, honouring the contributions of community and justifying the societal investment and engagement in research. However, for this power shift to be robust and meaningful, co-design must move beyond isolated pilot projects. It needs a commitment in project planning to sustainable implementation and translation, ensuring that the process of co-design leads to more tangible, equitable, and lasting outcomes in the community [5, 36].

## Conclusions

Co-design is a potentially essential research method for promoting disability inclusion, but it is at risk. As its popularity surges across sectors and disciplines, so too does the danger of it being reduced to a hollow buzzword [1–3, 7, 12, 13]. We must resist this dilution. Instead, co-design needs to be elevated as a meaningful and rigorous research practice. To do so, we have proposed priorities that encompass shared definitions and language, capacity building, and clear and mandated reporting using co-designed frameworks. These priorities are grounded in both methodological rigour and in the values and principles of disability inclusive research, which involves doing research with rather than on people with disability [1, 37, 38]. Frameworks for inclusive disability research, including co-design, highlight the importance of working towards mutual learning, relational ethics, and the redistribution of power in knowledge production [1, 37, 38, 39]. Embedding these principles into co-design with people with disability strengthens its potential to be inclusive and transformative. Education to build shared language and capacity among researchers will begin to address superficial, but sometimes onerous, engagement [2, 3, 8, 13–15] being understood as disability-led knowledge [3, 7, 8, 10, 11, 15, 19, 22, 23], and facilitate more impactful and workable outcomes [1, 11–17].

What gets reported is what gets valued and evaluated. Robust reporting structures are urgently needed to support the methodological integrity of co-design [2, 3], enhance transparency, and safeguard inclusive research against the drift toward tokenism [1, 2, 10, 11]. By increasing transparency in how co-design is described, evaluated, and disseminated, we can strengthen the credibility of inclusive research and ensure co-design delivers on its potential to shift power and create better solutions [1, 3, 11]. Mandating the use of reporting tools, incentivising high-quality engagement, and embedding accountability into funding and publishing systems are essential steps towards ensuring that co-design remains ethical, impactful, and authentic. However, we acknowledge that our recommendations are grounded in disability research occurring in the Global North. Co-design practices and challenges vary across geographic and disciplinary contexts, but future work can and should explore how these recommendations can be applied in diverse contexts in ways that are supportive and transformational.

We believe these developments can provide a platform for co-design to be differentiated from other participatory methods, preventing the term from being misused and diluted into an umbrella term for any level of patient, public, or partner involvement. Research that carries the label of co-design will be more likely to represent the perspectives of the co-designers. Co-design is an emancipatory and high level of engagement with the community that has the potential to be transformative and representative of community perspectives. Incorporating these suggestions into future research will help maintain rigour and safeguard the future of this critical research method.

## Data Availability

No datasets were generated or analysed during the current study.
